# Influence of Commercial Saturated Monoglyceride, Mono-/Diglycerides Mixtures, Vegetable Oil, Stirring Speed, and Temperature on the Physical Properties of Organogels

**DOI:** 10.1155/2014/513641

**Published:** 2014-09-21

**Authors:** Omar Gerardo Rocha-Amador, Jose Alberto Gallegos-Infante, Qingrong Huang, Nuria Elizabeth Rocha-Guzman, Martha Rocio Moreno-Jimenez, Ruben F. Gonzalez-Laredo

**Affiliations:** ^1^Unidad de Posgrado, Investigación y Desarrollo Tecnológico, Departamento de Ings Química y Bioquímica, Instituto Tecnológico de Durango, Bulevar Felipe Pescador 1830 Ote., Colonia Nueva Vizcaya, 34080 Durango, DGO, Mexico; ^2^Department of Food Science, School of Environmental and Biological Sciences, Rutgers University, The New Jersey State University, 65 Dudley Road, New Brunswick, NJ 08901, USA

## Abstract

The objective of this study was to evaluate the influence of gelator, vegetable oil, stirring speed, and temperature on the physical properties of obtained organogels. They were prepared under varying independent conditions and applying a fractional experimental design. From there a rheological characterization was developed. The physical characterization also included polarized light microscopy and calorimetric analysis. Once these data were obtained, X-Ray diffraction was applied to selected samples and a microstructure lattice was confirmed. Commonly, the only conditions that affect crystallization have been analyzed (temperature, solvent, gelator, and cooling rate). We found that stirring speed is the most important parameter in the organogel preparation.

## 1. Introduction

Gels have been described as materials that are “easier to recognize than define” [1]. Most of the times this problem comes from industry, which develops products with a gel name, just to be attractive to consumers [2]. However, gels have been accepted as semisolid materials comprising low concentrations (<15%) of gelator molecules to form a network self-assembly that entraps the solvent (in organogels both nonpolar components), preventing flow due to surface tension [3].

Gels can be defined both from a rheological behavior and from a structural feature. In a rheological point of view, a gel is a system that does not flow and has the presence of a plateau region of storage modulus and a low tan*δ* (<0.1) at an angular frequency from 10^−3^ to 10^2^ rad/s. The structural definition is based on the connectivity of the system. Gel is a system consisting of molecules, particles, and chains, which are partially connected to each other in a fluid medium by crosslinks to the macroscopic dimensions. Then the loss of fluidity is the result of connectivity. Both are operational definitions and may have the possibility of exclusions [2].

Organogels have been attracting much attention in biomedical and pharmaceutical fields, where the erosion of gels in stomach and intestines is important for drug delivery [4, 5]; therefore gels erosion has been applied for this purpose [6]. As oils are safe materials and are suitable for lipophilic components [7], they are considered a good option for organogels elaboration. That is why food industry is very interested in this type of systems as a replacement of hydrogenated fats [8]. Thus, understanding organogels, definition is closely related to their characteristics and their crucial potential to develop new applications.

Organogels microstructure is possible in part due to fat crystallization. This phenomenon has been widely studied in several scientific fields. The case of fat oils is very important due to their applications in food industry. The study of crystal nucleation, dissolution, and agglomeration in the overall precipitation scheme implies practical difficulties. Nucleation and crystal growth are spontaneous processes, which diminish the energy of the growing particle and nucleation process in order to overcome activation energy; that is, the critical cluster (critical size) is the cluster with maximum Gibbs free energy. The activation barrier may be represented by three parameters: supersaturation ratio, reaction temperature, and interfacial energy [9]. Following crystal formation in organogels can give detailed information on how their preparation is affecting their final structure.

The crystallization behavior is related to concentration of gelator [10], cooling rate [11, 12], and shear rate on fat crystals [13, 14]. Depending on the system, gels are formed by different forces and interactions and there are some parameters that affect, such as pH, ionic force, and mechanical forces. The plastic fat gels are formed due to the aggregation of fat crystals by several forces as van der Waals, dipole-dipole, hydrophobic, and hydrogen bond interactions between crystals [15]. These interactions also give gels a fractal behavior. Fractal is a geometric pattern that is repeated at every smaller scale to produce irregular shapes and surfaces [16].

Hence, since the study of organogels is very complex, it must include structural analysis and rheological and thermal behaviors in order to have a clear understanding on how preparation parameters are affecting the final product. Thus, our objective was to evaluate the influence of gelator (type and concentration), type of vegetable oil, stirring speed, and temperature preparation on the physical properties of obtained organogels.

## 2. Materials and Methods

### 2.1. Materials

Three different vegetable oils (soybean, canola, and corn oil) were used. They have important differences in composition of saturated fatty acids (SFA), monounsaturated fatty acids (MUFA), and polyunsaturated fatty acids (PUFA). The approximate compositions of soybean (SFA 15.65%, MUFA 22.78%, and PUFA 57.74%, Wesson Brand), canola (SFA 7.36%, MUFA 63.27%, and PUFA 28.14%, Wesson Brand), and corn (SFA 12.94%, MUFA 27.57%, and PUFA 54.67%, Mazola Brand) were considered. These oils were bought at a local supermarket in New Brunswick (New Jersey, USA). Myverol (mainly monoglycerides glyceryl monostearate 49%, glyceryl monopalmitate 48%, and calcium silicate 3%) and Myvatex (mixture of monoglycerides and diglycerides, soy monoglycerides 35–45%, stearic acid monoester with propane-1-2-diol 40–50%, sodium 2-stearoyl lactate 10–15%, and calcium silicate 3%) were provided by Kerry, Mexico/USA, and used as gelator agents.

### 2.2. Gels Preparation

Gelators Myverol (MV) and Myvatex (MX) at three different concentrations (8, 9, and 10%) were heated (70, 80, and 90°C) on glass containers until complete melting, and then either vegetable oil (soy (SY), canola (CA), or corn (CN)) was added. Samples were stirred (T 25 digital Ultra-turrax, IKA, North Carolina, USA) for five minutes at three independent speeds (3600, 7200, and 12000 rpm) to homogenize them; then, samples were cooled to room temperature and stored in refrigerator for 24 hours.

### 2.3. Rheological Tests

Dynamic frequency sweep tests were performed in a rheometer (ARES Rheometer 902-30004, TA instruments, New Castle, DE, USA) using parallel plates geometry (25 mm diameter) under the following experimental settings: strain 0.1%, temperature 20°C, frequency sweep 0.1–100 rad s^−1^, and a gap of 1 mm. The *G*′ and *G*′′ modules, complex viscosity, and delta tangent for each experimental sample were obtained.

### 2.4. Polarized Light Microscopy

Polarized light microphotographs of organogels were obtained using a polarized light microscope Linkam Brand (T95 System controller; T95 Linksys 32 soft, 2009, Tadworth, UK). It was equipped with a Q imaging camera (Color RTV 10 BIT) using an Olympus TH4-100 Halogen lamp power supply unit (LTS120 temp. controlled stage). Images were taken at 20°C, and video was taken using the Camtasia Studio V 7.0 (Build 1426, 2010, Techsmith Corporation, Okemos, MN, USA).

### 2.5. Differential Scanning Calorimetry

Thermal behavior of samples was studied using a differential scanning calorimeter (DSC Q2000 TA instruments, Schaumburg, Ill, USA) equipped with refrigerated cooling system (RCS 40 TA Instruments, Schaumburg, Ill, USA). Samples were heated at 120°C (30 min) for deleting fat thermal memory. Up to three cycles of heating were used depending on the experimental sample. Gelation temperature and melting enthalpy for each experimental sample were recorded.

### 2.6. X-Ray Diffraction

X-ray diffraction experiment was developed by the use of a PXRD conduced with a D/M-2200T automated system (Ultima Rigaku, Tokyo, Japan) with Cu K*α* radiation *λ* = 1.5406 A. Patterns were collected at 2*θ* angles of 3° to 50° at a scan rate of 2°/min. A graphite monochromator was used and the generator power settings were fixed at 40 kV and 40 mA. Spectra were analyzed with specialized software (Xpowder Pro Ver. 2010.01.30, Woburn, Massachusetts, USA) for clarifying peaks, applying a single functional filter smoothing and five cycles of Fourier deconvolution.

### 2.7. Fractal Dimension

The fractal dimension of the samples was calculated using (1) according to a described method [17], which is based on the Avrami relationship. Consider

(1) $$\begin{array}{c} \ln\left(1-X_{\text{cr}}\right)=-kt^{D}, \end{array}$$

where *X*_cr_ is the crystallinity of the system; *t* is time; *k* is a constant; and *D* denotes the dimension of growing. *D* and *k* were calculated by nonlinear estimation using Levenberg-Marquardt algorithm (Statistica v 7.0, Tulsa, OK, USA). Crystalline fraction of samples was calculated adjusting values from complex viscosity of the system removing complex viscosity of the solvent:

(2) $$\begin{array}{c} X_{\text{cr}}=\frac{\eta^{*}\left(t\right)-\eta }{\eta^{*}\left(\infty \right)-\eta }. \end{array}$$

### 2.8. Microstructural Parameters

A universal relation has been developed [18], but in the present work a previous relationship was used [19], supported by earlier theoretical work [20] in order to evaluate structural parameters. In this experimental work, a three-dimensional colloidal network was considered as being composed of interconnected flocs and the fractal dimension was used to quantify the relationship between the average floc size and the particle concentration of the colloidal network. The fractal nature of fat crystal networks has been already presented [21] and explained the power-law relationship followed by *G*′ and the solid fat content (SFC) data for fat samples with low solid fat content (<10%). Rheological data from experimental work can lead us to obtain a parameter related to elastic modulus *ϕ* (the solid fraction previously obtained by *X*_cr_), *λ*, and *m*, obtained by nonlinear estimations (Statistica v 7.0, Levenberg-Marquardt algorithm, Tulsa, OK, USA). One has

(3) $$\begin{array}{c} G^{\prime }=\lambda \phi^{m}. \end{array}$$

The Hamaker constant was determined following reported methodology [18, 22] where *A* is the Hamaker constant, *a* is the diameter of the particles within a floc, and *d* is the average distance between clusters. Microscopy images were analyzed using Corel Photo-paint X4 (Ver. 14.0.0.567, Menlo Park, CA, USA) applying channel separation (CMYK) in order to obtain floc and distance parameters as clearly as possible. Consider

(4) $$\begin{array}{c} \lambda \sim \frac{A^{3}}{\pi ad^{2}}. \end{array}$$

### 2.9. Statistical Analysis

Comparisons and calculations of parameters were obtained by nonlinear estimation with Statistica Software (Ver. 7.0, 2007, Tulsa, OK, USA) using Levenberg-Marquardt algorithm. Mean effects on interactions between processing parameters and fractal dimensions and microstructural parameters were determined by screening design (JMP Ver. 7.0, 2007, Cary, NC, USA).

## 3. Results and Discussion

### 3.1. Dynamic Rheology

**Figure 1:**
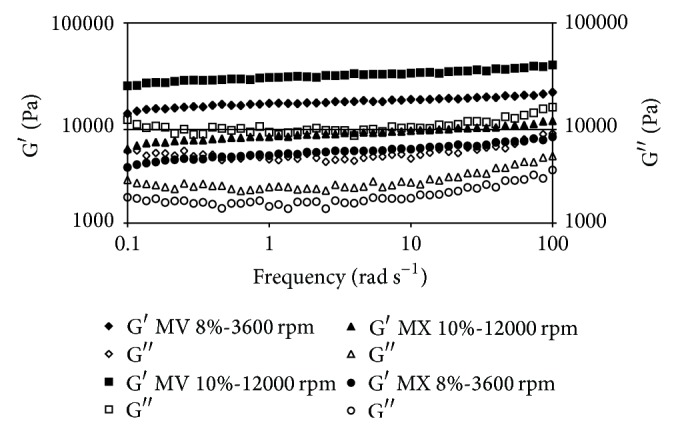
Frequency sweep comparison of canola oil samples. *G*′ module (filled), *G*′′ module (empty). Gelators used: Myverol (MV) and Myvatex (MX).

**Figure 2:**
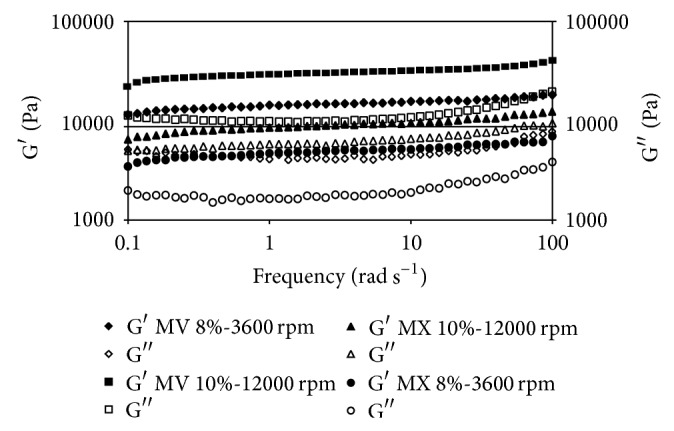
Frequency sweep comparison of corn oil samples. *G*′ module (filled), *G*′′ module (empty). Gelators used: Myverol (MV) and Myvatex (MX).

**Figure 3:**
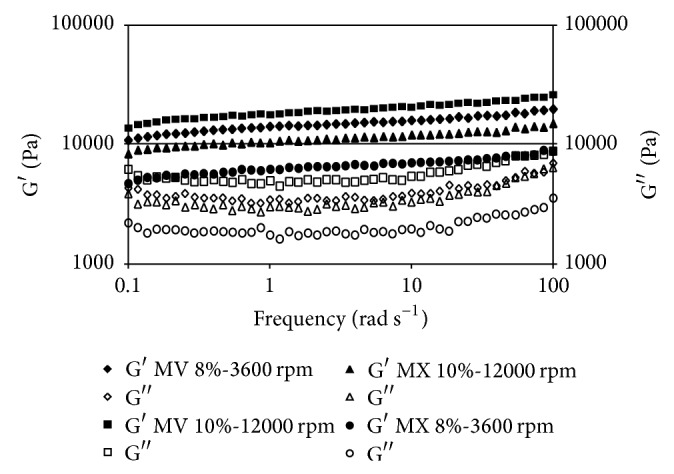
Frequency sweep comparison of soy oil samples. *G*′ module (filled), *G*′′ module (empty). Gelators used: Myverol (MV) and Myvatex (MX).

Oscillatory rheological data for organogels made with canola oil are shown in Figure 1. Higher values of *G*′ were observed for MV gels at higher frequencies used at 10% gelator concentration, 70°C, and 12000 rpm. The elastic module (*G*′) is associated with the solid behavior of the sample. Thus, this value may indicate that, at higher shear rates, structural order produced by lipid chains (i.e., acylglycerides) is organized in a more stable configuration, as a function of more contact between molecules that lower the necessary energy for self-organization and structure building. Similar findings have been reported previously [13]. Theoretical analysis of gels indicated that small particles could be aggregated to form large clusters with tenuous, chain-like, and self-similar structures that eventually span the entire enclosing space [23]. This behavior not only was influenced by shear rate, but also seems to be influenced by the type of gelator used as can be seen in Figures 1, 2, and 3. From these Figures it can be observed that at the same conditions MV organogels always give higher elastic modules in comparison with the MX. The mechanisms of aggregation of gelators in organogels occur primarily through van der Waals forces, specific intermolecular hydrogen bonding, electrostatic forces, *π*-*π* stacking, or London dispersion forces [24]. Thus, in function of the solvent and the gelator molecule, which type of intermolecular force predominates to stabilize the self-assembly primary structure, the growth mode, and finally the organogel microstructure and its thermomechanical properties is determined [25]. However, the relationship between gelator chemical structure and its gelling capability is not evident a priori in most cases [26]. However, it is desirable for gel modules to maintain a stable behavior during the complete frequency range. Solid behavior is predominant in all samples at the experimental shear range used, so it can be considered suitable for an excipient.

Similar behavior was observed in the organogels prepared with different oils (Figure 3), except for soy oil (SY), which seems to have a different effect, producing more elastic gels at the same conditions. SY seems to be favored by its higher solid fatty acids content, which gives more elastic modules to Myvatex gelator samples. An explanation of this behavior has been already reported [25]. In there, candelilla wax and amides derived from (R)-12-hydroxystearic acid were tested as gelators of safflower oil, observing that the increase in the hydrocarbon chain length raises both the organogel resistance to deformation and its instant recovery capacity. However, the extended recovery capacity of the gel decreased.

As can be seen in Figure 3, the behavior of each sample made with different oil at the same conditions changes significantly. This may be related to the different composition of vegetable oil and how each component is reacting at different preparation conditions. Although the higher elastic modules are obtained with canola (CA) and corn (CN) oil samples, in general CA gave the higher elasticity samples in comparison with those made with CN and SY. This behavior could be related to the monounsaturated fatty acids (MSFA) and polyunsaturated fatty acids (PUFA) contents. It appears that single unsaturation helps to join molecules into more stable structures.

### 3.2. Polarized Microscopy

**Figure 4:**
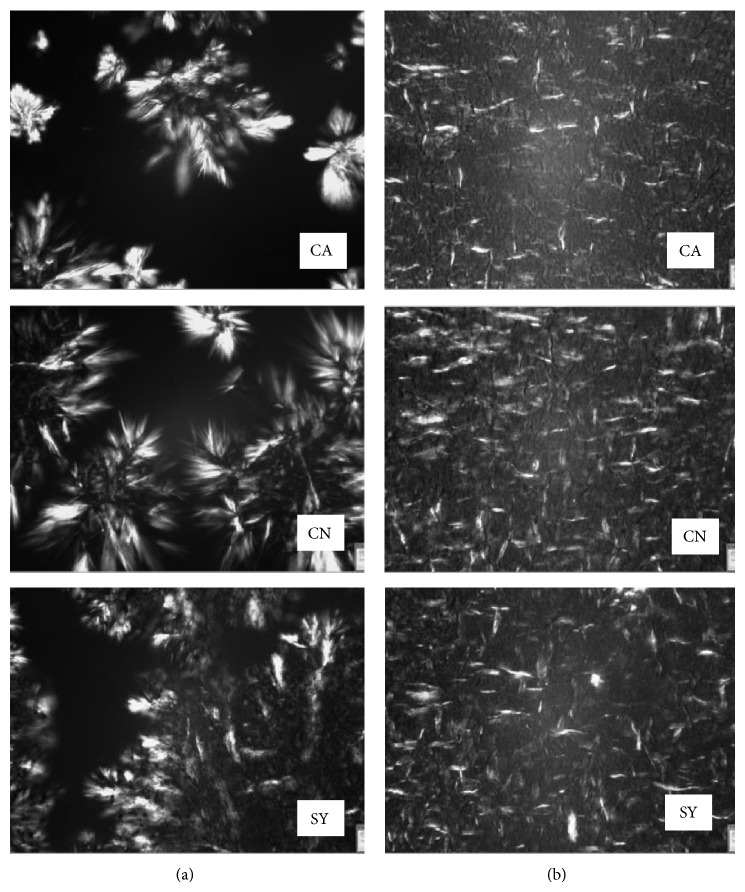
Gels prepared with canola (CA), corn (CN), and soy (SY) oils at concentration 9%, 7200 rpm, and 80°C of gelators Myvatex (a) and Myverol (b).

Obtained micrographs (Figure 4) showed that Myvatex tends to crystallize as spherulitic forms, some of them with larges spaces between these spherulites, while samples prepared with Myverol crystallized as fibrillar networks. So this could explain the higher elastic modules on samples prepared with this gelator. It was reported [10] that the higher number of hydroxyl groups related to the diacylglycerides was correlated with a lack of gelation into the organogels. This gives further evidence to the fact that hydrogen bonding is critical to the organogelation.

### 3.3. Differential Scanning Calorimetry

Statistical analysis of enthalpy data showed that gelator type and its concentration (*P* < 0.05) were the most important factors that influence enthalpy (Table 1). The statistical analysis showed a very complex phenomenon influenced by the following interactions: oil ∗ oil, concentration ∗ gelator, and gelator ∗ temperature. Organogels obtained with Myvatex gelator showed two different nucleation stages related to the presence of two main types of molecules (mono- and diacylglycerides); so probably distributed free energy makes gels tend to crystallize as spherulites rather than as a needle shaped network. However, not only the gelator is important, but also its concentration and several interactions including vegetable oil type and temperature are important. At this point, it is interesting to highlight that the temperature factor does not have influence on the enthalpy of organogels. Thus during cooling, both components might develop a mixed molecular packing with vegetable oils [27]. On the other hand, Myvatex gelator (MX) does not seem to be affected significantly by any preparation conditions. Finally, it is clear that the use of Myverol at higher concentrations produces organogels with higher values of gelation enthalpies, indicating more stable structures.

### 3.4. X-Ray Diffraction

A comparison of *d* spacing data was done in order to elucidate not only possible arrangements in packing, but also the most probable polymorphic forms in batches. Since they were complex mixtures of components (nonpure components), at least two different packings and polymorphs were selected. The first selection was the one with more intensity.

In Table 2 it can be noticed that most of the structures obtained are mainly *β* polymorphs, because most of them are amorphous materials and *b* crystals are present only in part. Although MX also gave a good structure, it was only possible under the highest energy conditions.

In order to compare different samples behaviors with a representative value, fractal dimension and microstructural parameters were obtained and presented below.

### 3.5. Fractal Dimension

Samples fractal dimensions (Table 3) were obtained from rheological behavior according to others [17], on which we should obtain crystalline fraction of each sample based on complex viscosity behavior and then get fractal parameter from nonlinear estimation. Once fractal data was obtained, ANOVA test was developed looking for some important influence. The model obtained was too complex and *t*-student was not sensitive enough to detect means without a significant error. Even in general no means were detected; some interesting effects were found when gelators and oils were blocked.

Several authors have used rheological determination of fractal dimensionality [23]. Also, several works on formation of organogels studied and correlated by Avrami exponent (*n*) with fractal dimensionality have been reported [17, 28]. However, it was indicated [29] that it may be inappropriate to compare Avrami derived fractal dimensionalities from several systems in which different nucleation mechanisms for gelators are involved. Thus, there are reports [17] on homogeneous nucleation with a fractal dimensionality near 2, while others [28] found a fractal dimensionality near 1 in a heterogeneous nucleation system. Thus in the present experimental work, fractal dimensionality was in that range (lower than 2), particularly when using mono- and diacylglyceride blends, indicating a possible heterogeneous nucleation process.

As an example, when the variable gelator (Myvatex) was blocked on canola oil (*P* = 0.047), the fractal dimension (*D*) was affected by the gelator concentration. Also, when corn oil temperature was blocked, the results indicated that higher temperatures render higher fractal dimensions. The fractal dimensions (*D*) determined by the particle counting method are sensitive to the spatial distribution of particles in the crystal network. Higher fractal dimensions occur in networks that are more ordered, whereas networks that arise from a more disordered nucleation and growth process result in lower fractal dimensions [22]. The fractal dimension value found in the present work was lower [17], similar [29], and higher [28] than other reports. Having greater fractal dimensions signifies more homogeneously distributed mass and/or more uniformly filled spaces [30].

Even though we did not find any significant mean on RPM versus *D* parameters, independently of the gelator, *D* fractal dimension tends to decrease as RPM increases. This could mean that as the shear rises there is an increase in disorder of the structure, which is contrary to reports from others [13, 14]. This could also indicate that different conditions are affecting gels structure and there is not necessarily a codependence on shear rate and order of the structure.

### 3.6. Microstructural Parameters

The *λ* parameter was obtained for each experimental condition (Table 3). The highest value of *λ* was found for Myverol (mainly monounsaturated fatty acids) in comparison with Myvatex. The gelator molecules are related to the nature of the short-range weak forces and the strong solvent dependence on the molecular self-assembling capabilities [31], so that molecules with higher molecular weight could affect crystal growth and its consequence on fiber interpenetration among vicinal spherulites.

However, not only gelator molecule is important, but solvent nature and interaction solvent-gelator are important too. In this way, experimental data was blocked for the two gelators in separated assessments. Results showed that Myverol is affected by the type of oil used for the sample (*P* = 0.0085), by the oil-concentration interaction (*P* = 0.04), and the concentration quadratic effect as well (*P* = 0.0072). Meanwhile for Myvatex, the oil effect was also important (*P* = 0.0766), but contrary to Myverol, it was affected by the oil quadratic (*P* = 0.43) and the temperature quadratic effects (*P* = 0.032).

Thermal parameters could be helpful to explain this behavior, because they are associated with the molecules polarity and the energy of the molecular interactions that are established with the crystal structure of gelators in their neat and organogel states [31]. Thus, the energy related to the organogel depends on the energy of the noncovalent interactions involved during the molecular self-assembly. As can be seen, higher enthalpies were observed for Myverol in comparison to Myvatex, like a structural parameter. Monoglycerides are known to be good initiators of fat crystallization and the matching in saturation and carbon chain length of both the gelator fatty acids and the oil phase are important in the particular lipid-lipid interactions [32].

For the Hamaker constant (Table 3), gelator appears as a main effect (*P* ≤ 0.0001), followed by the stirring speed (*P* = 0.052), as well as the interaction gelator-stirring speed (*P* = 0.088). This could indicate how stirring conditions are affecting molecular alignment. Also the bilayer packing seems to be favored by the particularities on CA oil composition of mainly the high MUFA and low PUFA contents. Most of samples analyzed for CN and SY oils were mainly indicating monolayer packings.

## 4. Conclusions

It was found that stirring speed affects significantly the structure of the organogels. Those prepared with Myverol are significantly affected by stirring conditions unlike Myvatex gels. Also the first gelator tends to form needle shaped network structures with better intermolecular forces according to their Hamaker constant and XRD analysis.

The stirring speed should be an important parameter to take into account, as well as the gelator and its concentration. It was found that temperature does not seem to be an important parameter in gels preparation. Finally, it is important to point out that most of the influence of the stirring speed conditions is apparently related to the oils higher MUFA and low PUFA contents.

## Figures and Tables

**Table 1:** Gelation enthalpy comparison for organogel samples.

Process conditions				Δ*H* g∗		
Gelator	(%)	Stirring 1/min	Temperature (°C)	Canola	Corn	Soy
				J/g	J/g	J/g
Myverol	8	3600	70	5.90 (0.18)	4.87 (0.30)	5.80 (0.17)
Myvatex	8	3600	90	5.72 (1.06)	4.54 (0.10)	2.38 (0.16)
Myvatex	8	12000	70	4.94 (0.33)	5.96 (0.08)	5.57 (0.04)
Myverol	8	12000	90	5.56 (0.06)	5.78 (0.46)	6.14 (0.26)
Myvatex	9	7200	80	5.38 (0.48)	4.26 (0.18)	7.40 (0.20)
Myverol	9	7200	80	7.04 (0.71)	6.52 (0.79)	6.83 (0.27)
Myvatex	10	3600	70	6.61 (0.11)	5.60 (0.60)	6.66 (0.15)
Myverol	10	3600	90	8.87 (0.25)	7.42 (0.30)	7.90 (0.45)
Myverol	10	12000	70	7.50 (0.35)	7.31 (0.54)	7.80 (0.46)
Myvatex	10	12000	90	5.87 (0.62)	6.28 (0.31)	5.79 (0.18)

^*^Mean (standard deviation).

**Table 2:** Comparison of lattice spaces of experimental organogels.

Oil	Gelator	[gelator] (%)	RPM 1/min	Temperature (°C)	*d* spacing at highest intensity (Å)						Probable packing		Probable polymorphic	
											Main	Secondary	Main	Secondary
CA	MV	10	12000	70	4.95	4.66	4.56	4.44	4.35		Monolayer	bilayer	*β*	*α*
CA	MV	10	3600	90	4.98	4.66	4.55	4.35			Bilayer	Monolayer	*β*	Sub-*α*
CA	MV	9	7200	80	4.58	4.49	4.35	4.12			Bilayer	Monolayer	*β*	Sub-*α*
**CN**	**MX**	**10**	**12000**	**90**	**4.58**	**4.48**	**4.38**	**4.12**			**Bilayer**	**Monolayer **	***β***	**Sub-*α***
CN	MV	10	12000	70	4.45	4.37	4.19	4.01	3.93		Monolayer	Bilayer	*α*	*α*
CN	MV	10	12000	90	4.98	4.61	4.35	4.13	3.91		Monolayer	Lamellar	*β*	Sub-*α*
CN	MV	9	7200	80	5.05	4.68	4.58	4.46	4.35	4.21	Monolayer	Bilayer	*β*	*α*
SY	MV	10	12000	70	4.63	4.54	4.38	4.13	3.97		Monolayer	Bilayer	*β*	*α*
SY	MV	10	3600	90	4.98	4.53	4.38	4.17	4.10		Monolayer	Bilayer	*β*	*β*′
SY	MV	9	7200	80	4.96	4.57	4.43	4.18	4.09	3.97	Monolayer	Bilayer	*β*	*α*

**Table 3:** Physical parameters of organogel samples.

Process conditions				Fractal			Structural parameter			Hamaker constant		
Gelator	(%)	Stirring 1/min	Temperature (°C)	Canola	Corn	Soy	Canola	Corn	Soy	Canola	Corn	Soy
Myverol	8	3600	70	1.62	1.53	1.54	16200	14500	11220	333	300	360
Myvatex	8	3600	90	1.56	1.61	1.58	4300	4300	5090	75	98	58
Myvatex	8	12000	70	1.52	1.51	1.60	3800	2800	4700	87	60	76
Myverol	8	12000	90	1.51	1.47	1.58	17000	11200	8900	404	126	195
Myvatex	9	7200	80	1.50	1.36	1.48	6600	2900	10500	93	57	96
Myverol	9	7200	80	1.57	1.57	1.56	21100	6250	21900	467	257	316
Myvatex	10	3600	70	1.65	1.49	1.68	6300	3100	9600	75	70	93
Myverol	10	3600	90	1.54	1.48	1.55	18500	15100	15850	406	205	425
Myverol	10	12000	70	1.48	1.67	1.50	30000	30500	13500	139	480	313
Myvatex	10	12000	90	1.61	1.59	1.57	7000	8050	7500	66	81	83
